# Oral microbiota: the overlooked catalyst in cancer initiation and progression

**DOI:** 10.3389/fcell.2024.1479720

**Published:** 2025-01-13

**Authors:** Xinlin Wang, Xin He, Bin Zhong

**Affiliations:** ^1^ Department of Respiratory Medicine, The First Affiliated Hospital of Gannan Medical University, Ganzhou, China; ^2^ The First School of Clinical Medicine, Gannan Medical University, Ganzhou, China; ^3^ Jiangxi Provincial Branch of China Clinical Medical Research Center for Geriatric Diseases, The First Affiliated Hospital of Gannan Medical University, Ganzhou, China

**Keywords:** oral microbiota, *Fusobacterium nucleatum*, *Porphyromonas gingivalis*, cancer, oncogenic mechanisms

## Abstract

The advancement of high-throughput sequencing technology in recent decades has led to a greater understanding of the components of the oral microbiota, providing a solid foundation for extensive research in this field. The oral microbiota plays an important role in an individual’s overall health. It has been shown to be significantly correlated with chronic human diseases, including diabetes, rheumatoid arthritis, cardiovascular disease, periodontal disease, and Alzheimer’s disease. Furthermore, tumor occurrence and development are closely related to the oral microbiome. Specific bacteria, such as *Fusobacterium nucleatum* (*F. nucleatum*), *Porphyromonas gingivalis* (*P. gingivalis*), *Streptococcus*, *Streptomyces*, *Prevotella*, and *Fibrophagy* gingivalis, play critical roles in cancer development. The oral microbiota has various oncogenic mechanisms, including bacterial inflammation, immunological suppression, tumor growth mediated by bacterial toxins, antiapoptotic activity, and carcinogenic effects. This paper reviews the role of the oral microbiota in the occurrence and progression of cancer and systematically elucidates the molecular mechanisms by which dysbiosis influences tumorigenesis and tumor progression. This information can provide a theoretical basis for exploring cancer treatment strategies and offer new insights for cancer prevention.

## 1 Introduction

Cancer is the second greatest cause of mortality worldwide. According to estimates from the World Health Organization, there were approximately 19 million new cancer cases globally in 2020, resulting in approximately 9.96 million deaths. The etiology of cancer is associated with various factors, including family inheritance, environmental factors, and metabolic damage (Flavahan et al., 2017). Since their discovery a few decades ago, an extensive amount of research has been conducted on carcinogenic bacteria. The significance of the microbiota is highlighted by the 16.1% global cancer risk related to microbial infections (Sun et al., 2020). Approximately 20% of tumors have a direct connection to particular viral or microbial infections. *Helicobacter pylori*, for example, has been shown to cause stomach cancer; hepatitis viruses are known to cause liver cancer; the Epstein‒Barr virus has been correlated with lymphoma and nasopharyngeal carcinoma; and the human papillomavirus is an important trigger of cervical cancer (Levrero and Zucman-Rossi, 2016; Volkova et al., 2021; El Tekle and Garrett, 2023). One of the most widely recognized instances of bacterial carcinogenesis is *Helicobacter pylori*. Through chronic infection, *Helicobacter pylori* can cause a variety of gastric malignancies, including gastric cancer (GC) and gastric lymphoma of mucosa-associated lymphoid tissue. Research has demonstrated that *Helicobacter pylori* can activate several signaling pathways and that it interacts intricately with host, environmental, and bacterial factors to promote carcinogenesis in the stomach mucosa (Wang et al., 2014). The bacterial protein Cytotoxin-associated gene A (CagA) can be translocated by *Helicobacter pylori* into human B lymphocytes and stomach epithelial cells. Translocated CagA is responsible for tyrosine phosphorylation, modulation of intracellular signaling pathways, and binding to Src homology 2 (SH-2). Moreover, translocated CagA inhibits apoptosis by activating extracellular signal-regulated kinase (ERK) and p38 mitogen-activated protein kinase (MAPK) in human B cells, thereby upregulating the expression of B-cell lymphoma2(Bcl-2) and Bcl-X (L). *Helicobacter pylori* directly transports CagA to B cells, where it is linked to the onset of Mucosa-associated lymphoid tissue lymphoma and other pathological processes (Lin et al., 2010). These data provide a theoretical basis for bacterial carcinogenesis.

As one of the largest microbial ecosystems in the human body, the oral cavity serves as the initial point of entry for the respiratory and digestive systems. It provides a habitat for a wide variety and abundance of microorganisms. More than a thousand different species of bacteria have been discovered in the oral cavity. Dental caries, gingivitis and other oral disorders can be caused by oral flora dysbiosis. It is also connected to other systemic disorders, such as liver cirrhosis, inflammatory bowel disease, diabetes mellitus, rheumatoid arthritis, and cardiovascular disease (Gao et al., 2018; Tuominen and Rautava, 2021) The connection between ecological dysregulation of the oral flora and cancer has drawn increasing attention as studies have progressed. Some bacteria, including *Streptococcus* species, *Streptococcus alimentarius* species, and *Prevotella* species, are closely associated with oral cancer. *Fusobacterium nucleatum* (*F. nucleatum*) and *Porphyromonas gingivalis* have been proposed as important promoters of colorectal cancer (CRC) and pancreatic cancer (Karpiński, 2019). These published findings provide evidence that the oral flora may be involved in cancer development and progression through multiple mechanisms. In this study, we discuss the processes through which dysbiosis of the oral flora contributes to the progression of cancer. A better understanding of these oncogenic mechanisms could lead to novel approaches for the detection and treatment of tumors.

## 2 Molecular mechanisms of *Fusobacterium nucleatum* in the development and progression of malignant tumors

### 2.1 Fusobacterium nucleatum

*Fusobacterium nucleatum* is a gram-negative, non-spore-forming, obligate anaerobe commonly found in the oral cavity. It belongs to the genus *Fusobacterium* and is a key pathogen in gingivitis and periodontitis (Han, 2015). As an opportunistic pathogen, *F. nucleatum* spans the gap between initial colonizers (e.g., *Streptococcus*) and anaerobic secondary colonizers (e.g., *Porphyromonas gingivalis*) and provides an anaerobic microenvironment for other anaerobes (Kolenbrander et al., 2010; Lamont et al., 2018). In addition to causing periodontal disease, *F. nucleatum* is often considered to be associated with the occurrence and development of oral cancer. In addition to inducing periodontitis, *F. nucleatum* is also frequently associated with the emergence and progression of oral cancer (Irfan et al., 2020). With the advancement of high-throughput sequencing technology, 16S rRNA has confirmed the existence of *F. nucleatum* in cancer tissues beyond the oral cavity (Brennan and Garrett, 2019). *F. nucleatum* can adhere to different locations by binding to and invading both epithelial and endothelial cells through its virulence factors, such as outer membrane protein A, *Fusobacterium* adhesin A (FadA), and Fibroblast Activation Protein-2(Fap2).

### 2.2 *Fusobacterium nucleatum* and colorectal cancer

**FIGURE 1:**
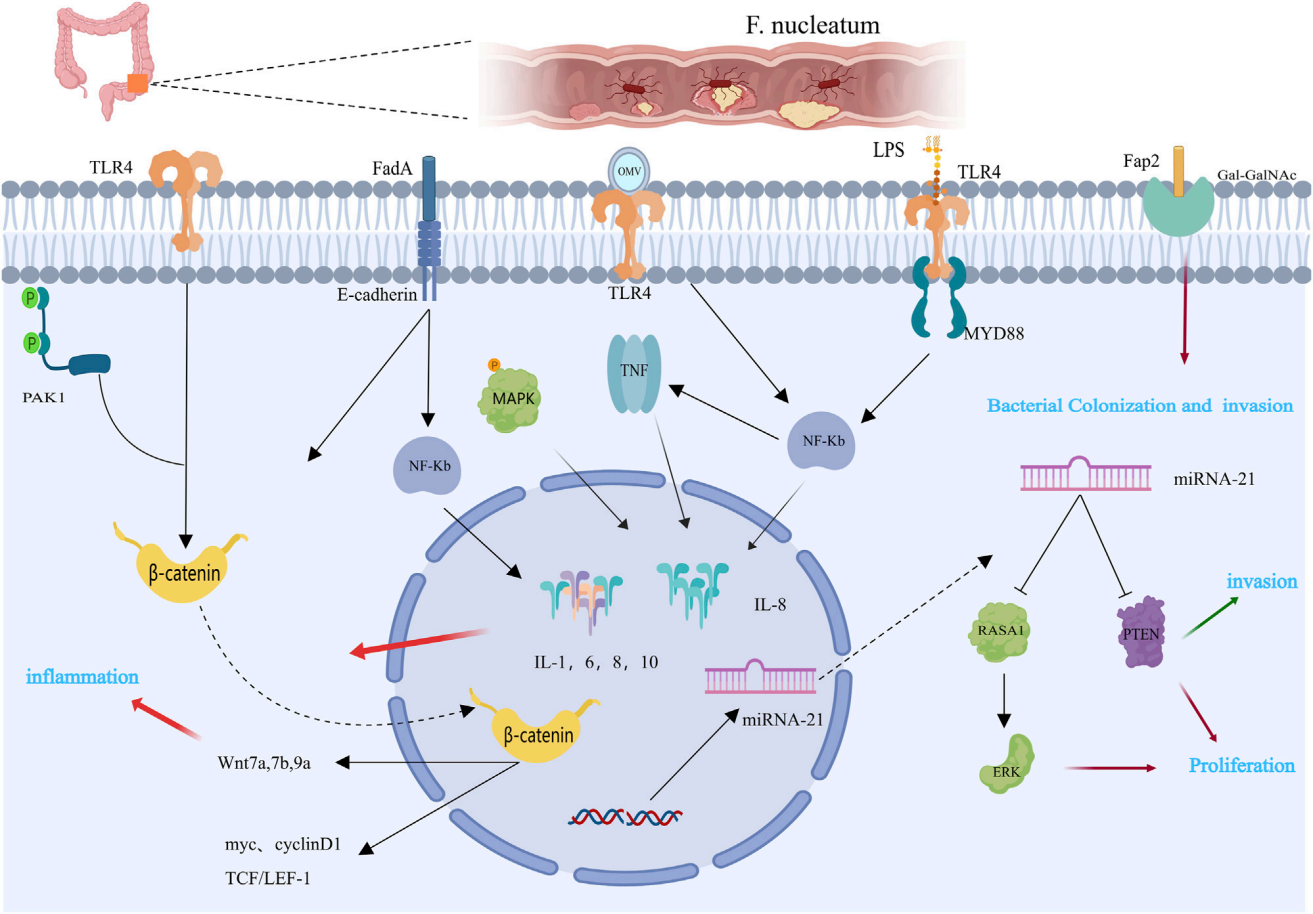
Mechanisms by which *Fusobacterium nucleatum* may contribute to colorectal carcinogenesis [This figure was created using MedPeer (www.medpeer.cn)]. The expression of β-catenin and NF-κB is mediated by the LPS of *Fusobacterium nucleatum* via TLR4. Additionally, through binding to E-cadherin, FadA promotes the Wnt/β-catenin pathway. By binding to the host factor Gal-GalNAc in CRC tissues, Fap 2 facilitates the nuclear colonization of *F. nucleatum*. The carcinogenic consequences of CRC are related to these pathogen-host interactions. OMV stimulates the production of pro-inflammatory cytokines, such as TNF and IL-8, and promotes intestinal inflammation by activating TLR-4 and affecting NF-κB signaling. By suppressing PTEN protein expression, miR-21 promotes proliferation and invasion of CRC cells.

Most colorectal cancer tissues contain *Fusobacterium nucleatum*, which is located primarily in the proximal colon (Mima et al., 2015; Yu et al., 2017). *Fusobacterium nucleatum* travels through the bloodstream from the oral cavity to other parts of the body, enriching the mucosal microbiota, adhering to and invading human endothelial and epithelial cells, leading to persistent cell infections, and ultimately exerting pathogenic effects outside the oral cavity (Chen et al., 2012; Pignatelli et al., 2023). We summarize the molecular pathways by which *F. nucleatum* promotes colorectal tumors (Figure 1). First, research indicates that *Fusobacterium nucleatum* primarily colonizes CRC tissues through the gastrointestinal tract, with a significant increase in the abundance of the virulence factor FadA in *F. nucleatum* (Wang and Fang, 2023). FadA promotes inflammation and participates in CRC development by activating two pathways. In the first pathway, FadA binds to E-cadherin on host epithelial cells, facilitating the internalization of FadA and activating β-catenin-regulated transcription, subsequently triggering the Wnt/β-catenin pathway. Activated β-catenin moves from the cytoplasm to the nucleus, increasing the expression of WNT signaling genes (such as wnt7a, wnt7b, and wnt9a), oncogenes (such as myc and cyclin D1), T-cell factors (such as TCF1, TCF3, and TCF4), and Lymphoid enhancer-binding factor 1 (LEF-1), leading to carcinogenic and inflammatory responses (Pignatelli et al., 2023; Wang and Fang, 2023). In another study, *F. nucleatum* was shown to participate in CRC by activating the β-catenin signaling pathway through the TLR4/P-PAK1/P-β-catenin pathway, suggesting that Toll-like receptor 4 (TLR4) and P21-activated kinases 1(PAK1) may be promising therapies for treating *F. nucleatum*-related CRC (Chen et al., 2017). Additionally, FadA promotes the expression of Nuclear Factor Kappa-B (NF-κB) genes, including numerous cancer genes, as well as genes that promote inflammation (Rubinstein et al., 2019). In another pathway, FadA activates the NF-κB signaling pathway, adhering to and invading human endothelial and epithelial cells, leading to increased levels of inflammatory cytokines such as Interleukin 6(IL-6), IL-8, IL-10, and IL-1β, especially IL-8, which are regulated by the p38 MAPK signaling pathway, tumor necrosis factor α(TNF-α), and NF-κB (Dharmani et al., 2011; McCoy et al., 2013; Quah et al., 2014; Rubinstein et al., 2019; Pignatelli et al., 2023; Wang and Fang, 2023). Second, Fap2 plays a crucial role in CRC development. Fap2 is a galactose-sensitive lectin and adhesin that is possibly involved in the virulence of *F. nucleatum* (Coppenhagen-Glazer et al., 2015; Fujiwara et al., 2020). Fap2 recognizes Gal-GalNAc, which is overexpressed in colorectal cancer, and mediates the colonization and invasion of *F. nucleatum* in CRC tumors. Abed et al. demonstrated that *F. nucleatum* injected intravenously colonized mouse colonic cancers in an Fap2-dependent manner through hematogenous pathways, indicating that host epithelial Gal-GalNAc receptors or targeting *F. nucleatum* Fap2 could reduce *F. nucleatum*-mediated enhancement of CRC (Abed et al., 2016). Additionally, Fap2 inhibits the interaction between immune cells (T cells and NK cells) and the T cell immunoglobulin and ITIM domain (TIGIT), reducing the activity of NK cells and T lymphocytes and thereby reducing the capacity of the immune system to combat cancer, leading to cancer cell growth and proliferation (Gur et al., 2015; Fujiwara et al., 2020; Stokowa-Sołtys et al., 2021). These findings provide new ideas for cancer treatment. The interaction between *F. nucleatum* and the TIGIT receptor hinders the receptor’s proper cancer-fighting function, suggesting that separating the receptor from other receptors might protect the immune system (Liu Y. et al., 2019). Third, like other gram-negative bacteria, *F. nucleatum* secretes outer membrane vesicles (OMVs), which are rich in envelope lipids and proteins, and plays significant roles not only in bacteria but also in interactions with host cells (Liu J. et al., 2019). OMVs stimulate host reactions to inflammation, facilitating the development of CRC. Biomolecules in OMVs interact with Toll-like receptor (TLRs), one of the molecular pathways involved in OMV participation (Kaparakis-Liaskos and Ferrero, 2015; Gerritzen et al., 2017; Sheikh et al., 2021). Depending on the bacterial species, OMVs can bind to TLRs to maintain homeostasis or promote disease progression. Engevik et al. demonstrated that OMVs activate TLR-4 in colonic epithelial cells, affecting NF-κB signaling, stimulating the production of TNF and IL-8 and encouraging gastrointestinal inflammation (Meng et al., 2023). OMVs induce immune tolerance in the tumor microenvironment (TME), indirectly promoting CRC progression. Interactions among different cells in the TME induce the expression of vascular endothelial growth factor and autocrine activation of its receptors, facilitating the proliferation of cells and angiogenesis (Barteneva et al., 2017; Meng et al., 2023). Small, noncoding RNAs named miRNAs control the expression of several target genes. Patients’ cancer diagnosis and course can be tracked via miRNAs as biomarkers (Farasati Far et al., 2023). miRNA-21 is a specific carcinogenic ncRNA of *Fusobacterium nucleatum* that is highly expressed in CRC (Yang et al., 2017; Wang et al., 2021). Multiple studies have shown that miRNA-21 regulates CRC cell proliferation, invasion, anti-apoptosis, and cell cycle progression by downregulating PTEN expression (Xiong et al., 2014; Yazdani et al., 2016; Wu et al., 2017). Y. Yang and colleagues proposed that *F. nucleatum* can trigger the TLR4/MYD88/NF-κB pathway, which in turn regulates the expression of miRNA-21 (Yang et al., 2017). Additionally, earlier studies have indicated that CRC progression is related to alterations in KRAS and RAS signaling. Ohta and colleagues confirmed that RASA1 is a direct target of miRNA-21. Their study demonstrated that inhibiting miRNA-21 leads to an increase in RASA1 expression (Ohta et al., 2009).

**FIGURE 2:**
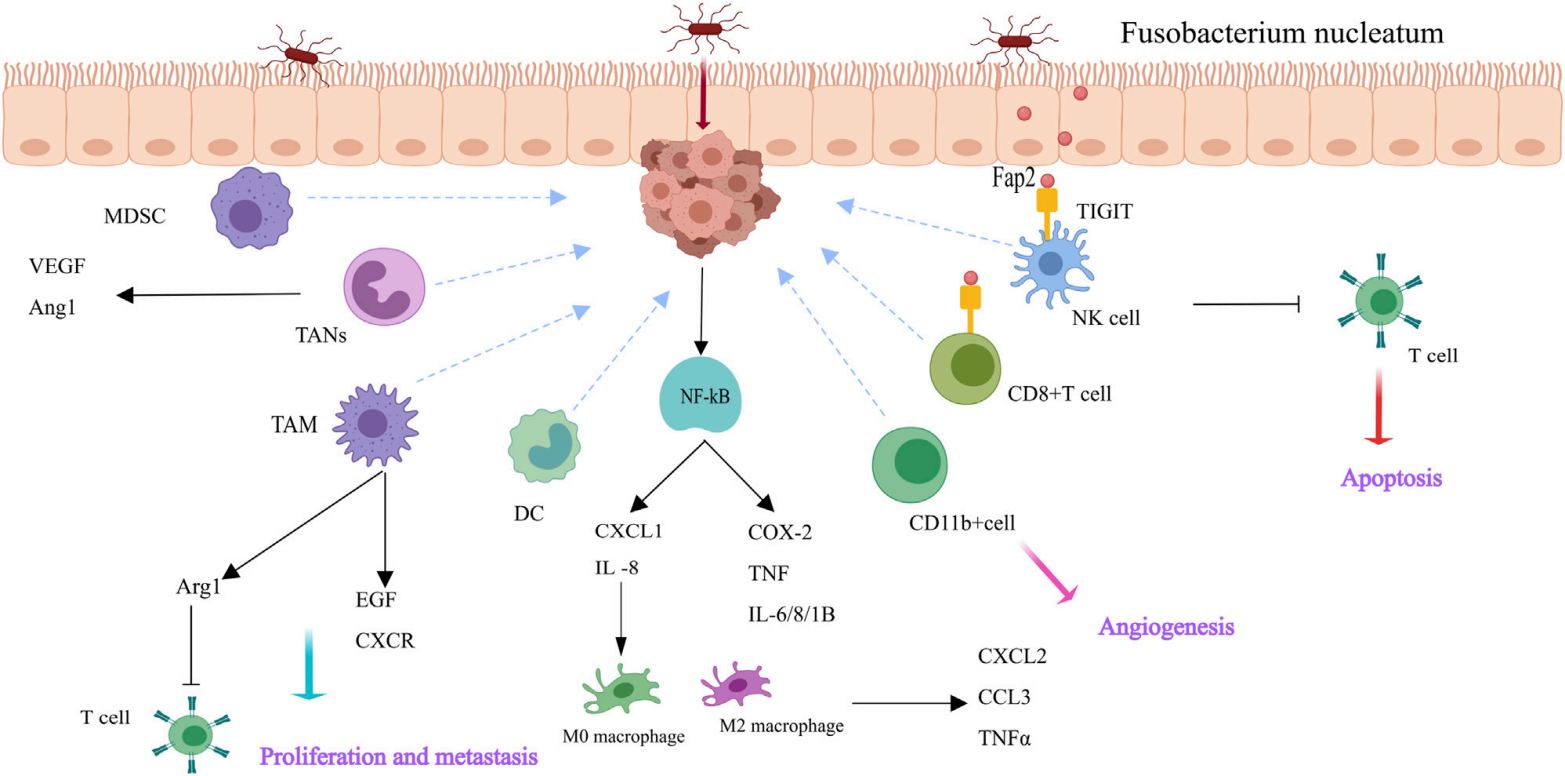
*Fusobacterium nucleatum* reshapes the tumor milieu in CRC cells and immune cells [This figure was created using MedPeer (www.medpeer.cn)]. F. nucleatum causes M2 macrophage polarization, upregulates pro-inflammatory cytokines, and activates the NF-κB pathway in CRC cells. Additionally, F. nucleatum attracts immune cells derived from bone marrow, including DC, TAN, TAM, and MDSC. TAM secretes a range of growth factors and chemokines that facilitate tumor proliferation, metastasis, and angiogenesis. *F. nucleatum* binds TIGIT (via Fap 2) and causes lymphocyte apoptosis, which suppresses the activity of human immune cells.

*Fusobacterium nucleatum* regulates intestinal tumors by first creating an inflammatory microenvironment and then downregulating adaptive antitumor immune responses. This selectively recruits myeloid-derived immune cells, such as myeloid-derived suppressor cells (MDSCs), tumor-associated neutrophils (TANs), dendritic cells (DCs), and tumor-associated macrophages (TAMs and M2-like TAMs), thereby constructing an immunosuppressive microenvironment (Kostic et al., 2013; Wang et al., 2021). Lymphocytes, including T, B, NK, and NKT cells, are the main immune cells in tumors. Antitumor cells include natural killer (NK) cells and cytotoxic CD8^+^ T cells (Chen et al., 2021). F. The nucleatum remodels the tumor immune microenvironment by increasing the number and function of immunosuppressive cells and inhibiting antitumor cells, thereby accelerating CRC progression (Figure 2). Kostic provided compelling evidence that exposure to *Fusobacterium nucleatum* in the Apc (Min/+) mouse model increases the tumor burden. *F. nucleatum* creates a proinflammatory microenvironment and infiltrates tumor immune cells, especially CD11b^+^ cells. Myeloid cells that are CD11b^+^ are crucial for angiogenesis and tumor growth (Coussens and Pollard, 2011; Kostic et al., 2013). TAMs generate proangiogenic substances and epidermal growth factors, which are mediators that promote tumor growth (Engblom et al., 2016). By secreting several growth factors and chemokines, M2-like TAMs not only suppress T cells by expressing arginase-1 but also promote tumor proliferation, metastasis, and angiogenesis (Arlauckas et al., 2018; Galdiero et al., 2018; Wang et al., 2021). *F. nucleatum* stimulates the NF-κB pathway in colorectal cancer cells, upregulating cytokines that promote inflammation and cause Chemokine (C-X-C motif) ligand (CXCL1) and IL-8 secretion. The tumor microenvironment is altered as a result of the recruitment of proinflammatory cytokines to nearby immune cells, which in turn encourage the release of their own cytokines (TNF-α, CXCL2, and CCL3) (Abed et al., 2016; Casasanta et al., 2020). Additionally, *F. nucleatum* induces adaptive immune responses. TIGIT is expressed by tumor-infiltrating lymphocytes, such as CD4^+^ and CD8^+^ T cells, as Gur and colleagues demonstrated. This receptor controls immunity mediated by T cells and is found on some T and NK cells. TIGIT is overexpressed on CD8^+^ TILs in cancer patients (Gur et al., 2015). Furthermore, *F. nucleatum* inhibits helper T cells and cytotoxic T lymphocytes through Fap2-mediated TIGIT interactions, inducing lymphocyte apoptosis and increasing tumor growth (Gur et al., 2015).

### 2.3 *Fusobacterium nucleatum* and esophageal cancer

**FIGURE 3:**
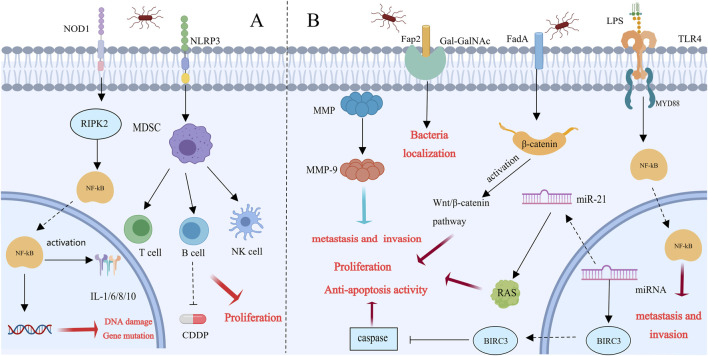
Possible molecular mechanisms of *Fusobacterium nucleatum* in tumor progression [This figure was created using MedPeer (www.medpeer.cn)]. **(A)** Esophageal dysbiosis promotes esophageal squamous cell carcinoma (ESCC) progression: NOD-RIPK 2 is activated when esophageal cells are infected by *Fusobacterium nucleatum*. Once NOD-RIPK 2 is activated, NF-κB is triggered, resulting in DNA damage and gene mutation. Furthermore, via activating NLRP3, *Fusobacterium nucleatum* generates MDSC. This environment may encourage tumor proliferation, metastasis, and chemical resistance, all of which can result in a poor prognosis. **(B)** *Fusobacterium nucleatum* accelerates breast cancer progression: *Fusobacterium nucleatum* promotes the colonization of breast cancer patients by identifying overexpressed GalNAc from Fap 2. In human breast cancer tissue, the entry of nucleated *Fusobacterium* cells into the underlying tissue was facilitated by the overexpression of MMP-9 and other matrix metalloproteinases. FadA adhesins also trigger the Wnt/β-catenin signaling pathway, which speeds up the development of breast cancer. Furthermore, *F. nucleatum* stimulates TLR4/MyD88 to enhance NF-κβ activation, which advances CRC. Breast cancer cell invasion and metastasis are facilitated by TLR 4/MyD 88 signaling. Furthermore, BIRC3 prevents apoptosis by blocking the caspase cascade, which causes tumors to develop and spread.

*F. nucleatum* is related to the progression of breast and colorectal cancer through the NF-κB, TLR4, IL-6, and TNF-α pathways (Chiang et al., 2023). Daichi Nomoto and colleagues injected TE-8 cells into nude mice with PBS or *F. nucleatum*. The tumor growth rate and weight of the cells treated with F. nucleatum significantly increased, indicating that the proliferation of cells that cause esophageal squamous cell carcinoma (ESCC) is significantly influenced by *F. nucleatum*. nucleatum promotes ESCC cell proliferation, metastasis, and chemoresistance by inducing DNA damage and gene mutations through the activation of the NOD1/RIPK2/NF-κB pathway. This activation may subsequently induce the expression of chemokines, proinflammatory cytokines, adhesion molecules, and other genes, potentially leading to cancer progression (Nomoto et al., 2022). Mengxia Liang and colleagues demonstrated that *F. nucleatum* induces the enrichment of immunosuppressive myeloid-derived suppressor cells by activating the PYD domains-containing protein 3(NLRP3) inflammasome. This activation leads to immune suppression, resistance to cisplatin treatment, and the promotion of the malignant proliferation of cancer cells. Targeting the elimination of *F. nucleatum* and preventing the accumulation of MDSCs could offer novel strategies and therapeutic approaches for treating esophageal squamous cell carcinoma (Liang et al., 2022). We summarizes the mechanisms related to breast cancer (Figure 3A).

### 2.4 *Fusobacterium nucleatum* and breast cancer

The most prevalent cancer among women globally is currently breast cancer. Early-stage patients have a cure rate of 70%–80%; however, secondary/metastatic late-stage breast cancer is currently considered incurable (Harbeck et al., 2019). Therefore, it is crucial to gain a deeper understanding of the factors that promote tumor progression, metastasis, and treatment resistance. This study demonstrated that intravenously injected *F. nucleatum* colonizes mouse tumor tissues in a Fap2-dependent manner and binds to Gal-GalNAc, which is highly expressed in colorectal cancer. The Fap2 outer membrane protein is the main Gal-GalNAc lectin for bacteria (Abed et al., 2016; Guo et al., 2024; Abed et al., 2016; Guo et al., 2024). Lishay Parhi et al. confirmed through a mouse model that *F. nucleatum* colonizes not only colorectal cancer but also human breast cancer tissues by recognizing Gal-GalNAc through Fap2, thereby stimulating breast cancer (BC) progression (Parhi et al., 2020). Additionally, Parhi reported that the expression of Matrix metalloproteinase 9 (MMP-9) is increased in *F. nucleatum*-cultured AT3 cells. MMP-9 is produced primarily by tumor cells and is involved in the invasion, angiogenesis, and metastasis of tumors. *Fusobacterium nucleatum* possesses highly virulent factors such as MMPs, which can facilitate its penetration into deeper tissues and stimulate the release of proinflammatory cytokines (Mehner et al., 2014; Guo et al., 2024). *F. nucleatum* promotes the formation and spread of tumors through the use of MMPs to invade tissues and release proinflammatory cytokines. In summary, *F. nucleatum* can colonize breast tissues through various pathways (Figure 3B). Like in colorectal cancer, β-catenin has been detected in breast cancer, indicating that *Fusobacterium nucleatum* accelerates the development of breast cancer through the adhesin FadA by triggering the Wnt/β-catenin signaling pathway (Guo et al., 2024). *Fusobacterium nucleatum* promotes CRC progression by stimulating TLR4/MyD88 to activate NF-κB (Van der Merwe et al., 2021). In breast cancer, a similar mechanism occurs in which bacterial lipopolysaccharides (LPSs) stimulate TLR4. The stimulation of TLR4 by bacterial LPS has the potential to induce inflammation, activate NF-κB, and produce antiapoptotic proteins (Rajput et al., 2013; Van der Merwe et al., 2021). LPS, by promoting MyD88 expression, contributes to breast cancer cell invasion and metastasis through autocrine or paracrine pathways (Yang et al., 2014). Additionally, the expression of microRNA-21 is increased by NF-κB, increasing RAS levels and promoting tumor cell proliferation. Another tumorigenic pathway involves NF-κB-mediated upregulation of BIRC3, which inhibits apoptosis by blocking the caspase cascade (Yang et al., 2017; Zhang S. et al., 2019; Guo et al., 2024).

An increasing number of studies indicate that *Fusobacterium nucleatum* plays a role in the development of different kinds of tumors. The mechanisms by which *F. nucleatum* leads to tumor occurrence and development include enhanced proliferation, invasion, and antiapoptotic effects; the establishment of a tumor-promoting immune environment; and the induction of chemotherapeutic drug resistance. *F. nucleatum*’s distinct virulence factors may function as effector molecules, converting healthy epithelial cells into tumor cells. These compounds may offer novel medications, therapeutic intervention targets, and approaches. Ongoing research into these carcinogenic mechanisms aims to provide further evidence for cancer diagnosis and treatment.

## 3 *Porphyromonas gingivalis* plays an important role in malignant tumors

### 3.1 Porphyromonas gingivalis

*Porphyromonas gingivalis* (*P. Gingivali*) is a common oral pathogen and a key causative agent of chronic periodontitis. Recent evidence suggests that *P. gingivalis* is not only an opportunistic pathogen of the oral mucosa and a major component of the oral microbiota but also a significant mediator of the development of various seemingly unrelated cancers, such as oral cancer, pancreatic cancer, and gastrointestinal cancer. The mechanisms by which *P. gingivalis* promotes the growth of tumors have gradually come to light in recent years.

### 3.2 *Porphyromonas gingivalis* and oral cancer

**FIGURE 4:**
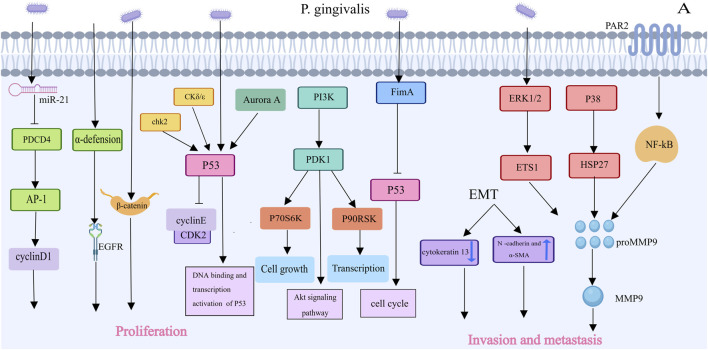
Porphyromonas gingivalis’s Contributions to Oral carcinoma of squamous cells Proliferation and Invasion [This figure was created using MedPeer (www.medpeer.cn)]. Porphyromonas gingivalis inhibited the level and activity of the p53 tumor suppressor, increased activated PI3K/Akt,and promoted OSCC proliferation via the miR-21/PDCD4/AP-1 axis. Porphyromonas gingivalis increased MMP expression and triggered epithelial-mesenchymal transition, which facilitated oral cancer cells’ invasion and metastasis.

Previous studies have shown that *Porphyromonas gingivalis* can promote tumor progression by affecting multiple signaling pathways. Tumor cells have the ability to proliferate indefinitely and uncontrollably (Figure 4). Thus, enhanced bacterial proliferation is a characteristic that promotes tumorigenesis, which is particularly evident with *P. gingivalis*. Through a variety of methods, *P. gingivalis* has been shown in certain *in vitro* experiments to promote the proliferation of (oral squamous cell carcinoma) OSCC lines. For example, in a model in which *P. gingivalis* infects OSCC TCA8113 cells, the expression of its target genes cyclin D1 and activator protein 1 increased in infected cells. Additionally, *P. gingivalis* increased OSCC proliferation by upregulating the expression of cyclin D1, a critical regulator of cell proliferation, through the miR-21/PDCD4/AP-1 axis. When the expression of programmed cell death 4 (PDCD4) is downregulated, miR-21 expression increases (Chang et al., 2019; Li et al., 2023). *Porphyromonas gingivalis* can regulate cyclins; reduce the levels and activity of p53 tumor suppressors via kinases such as Chk2, Aurora A, CK1δ, and CK1ε; and activate the PI3K/Akt pathway, encouraging the growth of primary gingival epithelial cells through the S phase of the cell cycle. In a way dependent on fimbriae (FimA), *P. gingivalis* stimulates the proliferation of gingival epithelial cells (GECs), where long FimA fimbriae are responsible for activating genes associated with the cell cycle and proliferation (Kuboniwa et al., 2008). *P. gingivalis* can also induce the activation of noncanonical β-catenin via proteolytic dissociation of the β-catenin destruction complex. *P. gingivalis* may induce β-catenin activation in epithelial cells, which could lead to a proliferative phenotype (Zhou et al., 2015). Additionally, bacteria can increase α-defensin expression, and the binding of α-defensin to the receptor for the epidermal growth factor signaling pathway promotes the development of cancer cells (Hoppe et al., 2016; Li et al., 2023). Further research is needed to confirm whether these mechanisms are related to *P. gingivalis* infection in oral squamous cell carcinoma cells.

Another characteristic of malignant tumors is invasion and metastasis, which involve the process of cancer cells spreading throughout the body (Figure 4). *Porphyromonas gingivalis* can increase the expression of matrix metalloproteinases and initiate epithelial‒mesenchymal transition (EMT), which can facilitate the invasion and metastasis of oral cancer cells. Gingivalis induces the creation of the MMP9 precursor, which is then activated into MMP9, by regulating the ERK 1/2-ETS 1, p38/HSP 27, and PAR2/NF-κB pathways (Li et al., 2023). MMPs primarily degrade the extracellular matrix and basement membrane, which allows oral cancer cells to invade and spread (Li et al., 2023). *P. Gingivalis* also induces EMT, enhancing the production of MMP-1 and MMP-10 (Chattopadhyay et al., 2019). When OSCC cells are repeatedly infected with *P. gingivalis*, they transform into a mesenchymal-like phenotype, with decreased expression of epithelial markers (such as cytokeratin 13) and increased expression of mesenchymal biomarkers (such as N-cadherin and α-SMA) (Ha et al., 2015; Li et al., 2023).

**FIGURE 5:**
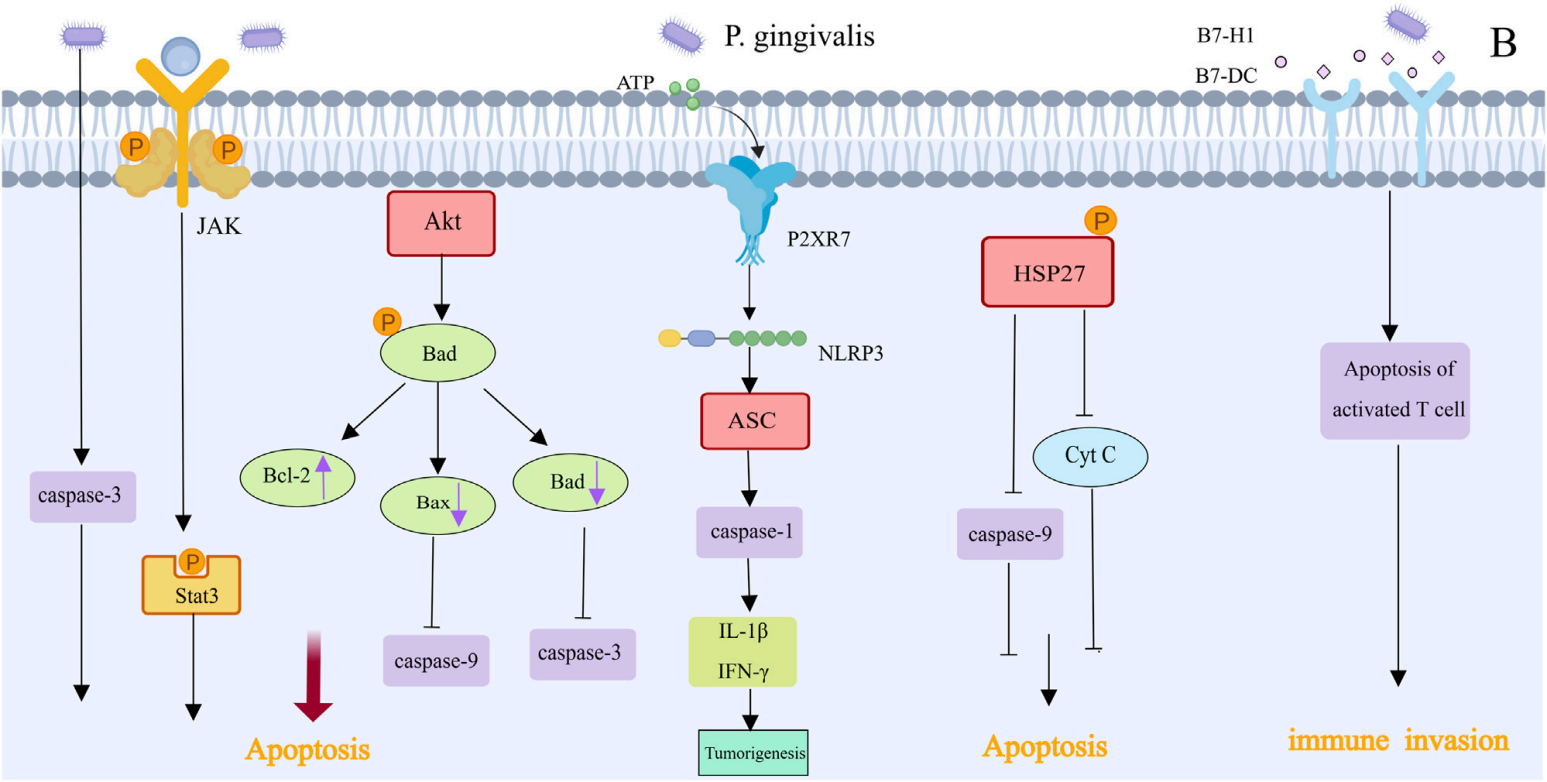
Apoptotic effect and immune invasion of Porphyromonas gingivalis in OSCC [This figure was created using MedPeer (www.medpeer.cn)]. Numerous anti-apoptotic pathways are activated by infection with Porphyromonas gingivalis. Bad mediated the mitochondrial release of cytochrome c and promoted apoptosis. The JAK1/Akt/Stat3 signaling pathway was activated to control the intrinsic death pathway of mitochondrial cells, and it was possible to inhibit the apoptosis of gingival epithelial cells by blocking the activation of effector protein caspase-3. Furthermore, apoptosis is inhibited by the activation of the P2RX7 receptor, cytochrome C release inhibition, and HSP27-mediated caspase-9 activation. Certain strains of P. gingivalis have the ability to induce T cell apoptosis and upregulate the expression of B7-H1 and B7-DC receptors, which may aid in the immune system’s escape from oral cancer.

Programmed cell death, or apoptosis, is an important mechanism to protect host cells from unlimited growth (Figure 5). *Porphyromonas gingivalis* infection can activate various antiapoptotic pathways to promote tumor growth. gingivalis prevents the activation of the effector protein caspase-3, which suppresses the chemically induced death of gingival epithelial cells. It can also control the intrinsic mitochondrial death pathway by activating the JAK1/Akt/Stat3 signaling pathway (Mao et al., 2007). Bad is a proapoptotic protein in the Bcl-2 family that mediates mitochondrial release of cytochrome c and promotes apoptosis (Yao et al., 2010). Akt mediates the phosphorylation of proapoptotic Bad in GECs infected with *P. gingivalis*, increasing Bcl-2 expression and decreasing the expression of proapoptotic factors such as Bax and Bad. This inhibits the action of downstream caspases, such as caspase-9 and caspase-3, by shifting the ratio of these protein interactions in favor of mitochondrial membrane integrity and resistance to apoptosis (Lamont et al., 2022). Nucleoside diphosphate kinase, which acts as an ATP, is an effector protein produced intracellularly by *P. gingivalis* that prevents apoptosis by consuming ATP and activating the purinergic P2X7 receptor. This disrupts NLRP3/ASC/caspase-1 inflammasome activation, inhibiting the secretion of Interferon-γ(IFN-γ) and IL-1β by CD8^+^ T cells and thereby accelerating the growth of tumors (Aymeric et al., 2010; Yao et al., 2010; Almeida-da-Silva et al., 2016; Wang et al., 2024). Additionally, phosphorylated heat shock protein 27 (HSP27) inhibits the release of cytochrome c and caspase-9 activation, delaying apoptosis (Chattopadhyay et al., 2019). In addition to inhibiting the intrinsic apoptosis of invading epithelial cells, *P. gingivalis* can promote the progression of the S phase of the cell cycle by inhibiting the p53 tumor suppressor gene through FimA (Wang et al., 2024). Tumor immune escape, which involves a highly complex mechanism, is one of the ten key features required for tumor incidence and progression (Figure 5). *In vitro* experiments revealed that after *Porphyromonas gingivalis* infection, the receptors B7-DC and B7-H1 were upregulated, with the expression of mRNAs in infected cells being higher than that in uninfected cells by 8.6-fold (B7-DC) and 6.4-fold (B7-H1). These results indicate that *P. gingivalis* strains can upregulate B7-H1 and B7-DC receptor expression in OSCC cell lines, leading to T-cell apoptosis, which may promote immune escape in oral cancer (Groeger et al., 2011). The increased expression of the B7-H1 receptor in host cells may also affect inflammatory diseases. Additionally, through the production of myeloid-derived suppressor cells, *P. gingivalis* undermines immunological surveillance, similar to that related to colorectal tumorigenesis, which is not described here.

### 3.3 *Porphyromonas gingivalis* and pancreatic cancer

**FIGURE 6:**
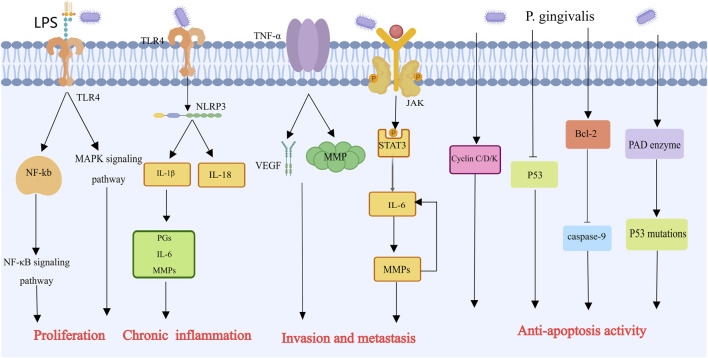
Mechanisms by which Porphyromonas gingivalis contributes to the progression of pancreatic cancer [This figure was created using MedPeer (www.medpeer.cn)]. In order to stimulate NF-kB and MAPK signals in immune cells and encourage the growth of pancreatic tumors, LPS can bind to TLR4. In addition, LPS can activate NLRP3, which facilitates the production of MMPs and inflammatory cells and is linked to the invasion and spread of tumors. In order to combat apoptosis, Porphyromonas gingivalis stimulates cyclin/cyclin-dependent kinase (CDK) and suppresses the p53 tumor suppressor. Furthermore, inhibition of caspase 9 and Bcl-2 inactivation were both encouraged by Porphyromonas gingivalis and prevented apoptosis.

With a mere 8% 5-year survival rate, pancreatic cancer is the most dangerous malignancy in the world (Siegel et al., 2018). Studies have demonstrated a correlation between alterations in the salivary microbiota and pancreatic cancer. Compared with healthy controls, patients with pancreatic cancer presented an increase of one species/cluster and a decrease of 25 species/clusters in their salivary microbiome. The major bacterial species detected in the saliva samples belonged to five different phyla: Firmicutes, Proteobacteria, CFB group bacteria, and Actinobacteria. Analysis with Human Oral Microbe Identification Microarray arrays revealed the involvement of bacteria in pancreatic diseases, suggesting a causal relationship between pancreatic cancer and the salivary microbiome of patients (Farrell et al., 2012). In a prospective study, people who had high levels of antibodies against *P. gingivalis* were twice as likely to develop pancreatic cancer than people who had low levels of antibodies. On the other hand, compared with those with low levels, those with high levels of antibodies against common oral bacteria had a 45% decreased risk of pancreatic cancer. This finding suggests two things: first, periodontal disease may increase the risk of pancreatic cancer, and antibodies against *Porphyromonas gingivalis* are the best markers for pancreatic cancer; second, raising antibody levels against particular oral symbiotic bacteria that prevent harmful bacteria from growing may lower the risk of pancreatic cancer (Michaud et al., 2013). Additionally, research by Ahn et al. revealed an association between periodontal pathogens such as *P. gingivalis* and the mortality rate of digestive cancers (Ahn et al., 2012). How *P. gingivalis* mediates the development of pancreatic cancer will be elucidated below (Figure 6). Experiments have shown that under hypoxic conditions, *P. gingivalis* enhances Pancreatic ductal adenocarcinoma cell proliferation, which is linked to bacterial survival, Akt signaling pathway activation and cyclin D1 expression. Previous studies have indicated that *P. gingivalis* promotes cancer cell growth through a TLR2-dependent mechanism. However, blocking TLR2 did not reduce *P. gingivalis*-induced PDAC cell proliferation, as Pancreatic ductal adenocarcinoma cell lines do not express TLR2. Thus, TLR2 is not a mediator of *P. gingivalis* growth-promoting action on pancreatic cancer cells (Gnanasekaran et al., 2020). Ochi et al. reported that LPS can activate TLR4 in mouse models of pancreatic cancer, leading to the activation of the NF-kB and MAPK signaling pathways in immune cells and promoting tumor development (Ochi et al., 2012). TLR4 also induces the expression of the NLRP3 inflammasome, which releases IL-1β and IL-18 (Hoque and Mehal, 2015). IL-1β stimulates the release of prostaglandins, IL-6, and MMPs, promoting angiogenesis, tumor metastasis, and invasion (Hou et al., 2003). IL-6 is a significant contributor to tumor growth and progression. The Jak1/Akt/Stat3 signaling pathway is triggered by *P. gingivalis*, which induces IL-6 to participate in gene expression for cell proliferation and survival. IL-6 also regulates MMPs, with increased MMP expression leading to elevated IL-6 levels, creating a positive feedback loop that exacerbates tumor development and metastasis (Mao et al., 2007; Tang et al., 2011; Chang et al., 2013). *P. gingivalis* also activates cyclins/cyclin-dependent kinases (CDKs) and inhibits the tumor suppressor p53 to prevent apoptosis. Furthermore, by promoting the inactivation of apoptotic Bcl-2 and inhibiting caspase-9, *P. gingivalis* activates the antiapoptotic protein Bcl-2 (Yao et al., 2010).

*Porphyromonas gingivalis* is a periodontal infection that has been linked to the emergence of several malignancies. Gingivalis promotes tumor progression through the following pathways: Promotion of cell proliferation: first, *P. gingivalis* can increase the proliferation of infected cells; second, *P. gingivalis* facilitates the invasion and metastasis of infected cells; third, *P. gingivalis* activates multiple antiapoptotic pathways to promote tumor development; fourth, *P. gingivalis* modulates the immune microenvironment to facilitate tumor progression. *P. gingivalis* may be linked to stomach and pancreatic malignancies. This implies that systemic carcinogenic effects can be caused by the oral microbiota, bacterially produced effectors, cells associated with inflammation, and mediators that can travel via saliva and blood to distant places. Importantly, there may be a direct relationship between *Porphyromonas gingivalis* and oral-gastrointestinal cancers. The carcinogenic effects of the oral microbiome might result from the secondary invasion of oral bacteria beyond the oral cavity but still within anatomically contiguous regions. Larger cohorts, greater controls, and other molecular epidemiology and mechanistic research will be essential in addressing concerns regarding potential problems that may arise in other areas.

## 4 Abundance of *Prevotella* influences the occurrence and development of malignant tumors

### 4.1 Prevotella

A crucial part of the oral microbiota, anaerobic bacteria, are the foundation of oral environmental balance. The phylum Bacteroidetes is one of the primary phyla, with *Prevotella* being the largest genus. *Prevotella* is recognized as one of the core genera of gram-negative anaerobic bacteria (Dewhirst et al., 2010). Over the past 2 decades, *Prevotella* has been extensively studied, but its clinical relevance remains limited. *Prevotella* species originating from the oral cavity appear to play significant roles in the gastrointestinal and respiratory tracts. Oral *Prevotella* species enter the gastrointestinal tract through saliva swallowing and continuously enter the lower respiratory tract through microaspiration. They possess antibacterial properties but can also act as potentially harmful substances on mucosal surfaces (Könönen and Gursoy, 2021).

### 4.2 *Prevotella* and head and neck cancer

The oral mucosal epithelium is the primary source of head and neck squamous cell carcinoma (SCC), pharynx, and larynx. In specific mucosal regions, such as the gums, tongue, and floor of the mouth, significantly higher levels of *Prevotella* intermedia have been identified in tumor sites than in healthy controls (Yang et al., 2021). Divya Gopinath reported notable differences in the bacterial composition between SCC patients and control subjects, with significant variations between the bacterial communities on the tumor surface and in deeper tissues. *Prevotella* species are predominantly found in deeper tissues, and their increased abundance aligns with previous reports, indicating a potential role in cancer progression (Gopinath et al., 2021). At the species level, the abundances of *Prevotella salivae*, *Prevotella loescheii*, and *Prevotella* intermedia are significantly greater in OSCC, suggesting a potential association between these bacteria and OSCC (Perera et al., 2018; Yang et al., 2021). S. Kageyama’s team used quantitative Polymerase Chain Reaction analysis and 16S rRNA gene amplicon sequencing to examine the bacterial composition and density in pre- and postoperative saliva samples from tongue cancer patients. They confirmed a significant reduction in the proportion of *Streptococcus*, pigment-producing *Prevotella*, and other *Prevotella* species in postoperative saliva (Kageyama et al., 2020). Further studies in laryngeal cancer patients revealed that the most prominent genera in the throat microbiota were *Streptococcus* (37.3%), *Prevotella* (10.6%), and *Fusobacterium* (11.3%). Significant differences in the relative abundance of bacterial genera between throat cancer patients and vocal cord polyp patients revealed that particular microorganisms may be related to throat cancer (Gong et al., 2014). Subsequent research on changes in throat microbiota characteristics before and after laryngeal squamous cell carcinoma (LSCC) resection surgery, as well as at 1 week and 24 weeks postsurgery, supports this view. The throat microbiota may be linked to human cancer signaling pathways, providing new molecular mechanisms to improve laryngeal cancer treatment and promote related research (Hsueh et al., 2020).

### 4.3 *Prevotella* and digestive tract cancers

For a long time, the upper gastrointestinal tract from the pharynx to the esophagus was believed to be free of microbiota until advanced molecular detection methods challenged these early beliefs. In 2004, Pei Z reported that the esophageal microbiota comprises six main phyla, *Firmicutes*, *Bacteroides*, *Actinobacteria*, *Proteobacteria*, *Fusobacteria*, and TM7, similar to the composition of the oral microbiota (Pei et al., 2004). In the esophagus, squamous cell carcinoma (SCC) is predominant, and *Prevotella* species are potential prognostic indicators for this cancer type (Liu et al., 2018). The association between the oral microbiota and the risk of ESCC was investigated in a case‒control study. The study assessed oral bacterial profiles from mouthwash samples of ESCC patients and controls and revealed a significant presence of *Prevotella*, especially *Prevotella nanceiensis*, in ESCC patients (Peters et al., 2017). Furthermore, an increased abundance of *Prevotella* was observed in adenocarcinoma mucosa and Barrett’s esophagus. Patients with lymph node metastasis had higher levels of *Prevotella* and *Helicobacter* than did those without metastasis. An increased abundance of *Prevotella* and *Streptococcus* was associated with poor survival, indicating a worse prognosis (Lopetuso et al., 2020).

Gastric cancer is one of the five most common gastrointestinal cancers in the world. Among the five most prevalent gastrointestinal malignancies worldwide is gastric cancer, along with colorectal cancer, pancreatic cancer, and esophageal cancer. *Helicobacter pylori* has long been recognized as a major pathogenic bacterium responsible for various gastric and duodenal diseases. In recent years, with the introduction of bacterial 16S rDNA identification techniques, research on the gastric microbiota has increased. Studies conducted in the United States and Hong Kong on the gastric microbiota of patients with gastric diseases have revealed bacterial phyla similar to those of the oral microbiota, specifically *Proteobacteria*, *Firmicutes*, *Bacteroidetes*, *Actinobacteria*, and *Fusobacteria*, which are consistent with the observed gastric microbiota across different populations (Bik et al., 2006; Li et al., 2009). In cardiac cancer tissues, the relative abundance of the genus-level bacteria *Streptococcus*, *Prevotella*, *Veillonella*, *Haemophilus*, and *Neisseria* has increased (Shao et al., 2019). Another cohort study analyzed differences between chronic gastritis patients and gastric cancer patients and revealed a significant increase in the numbers of *Streptococcus*, *Prevotella*, *Fusobacterium*, *Citrobacter*, *Clostridium*, and *Rhodococcus* bacteria in gastric cancer patients (Ferreira et al., 2018). At the species level, an increase in *P. melaninogenica*, *S. pharyngis*, and *P. gingivalis* populations in tumor tissues, whereas *H. pylori* and *P. gingivalis* populations significantly decreased (Liu X. et al., 2019). However, determining whether these enriched bacteria act as driving factors for carcinogenesis still requires further elucidation.

## 5 *Veillonella* is a biological marker for identifying lung cancer

### 5.1 Veillonella

*Veillonella* is a type of gram-negative, obligate anaerobic coccus. It is an early colonizer in the formation of oral biofilms and is found in high abundance within the oral microbiome. Currently, seven species of *Veillonella* have been identified in the oral cavity, including *Veillonella parvula*, *Veillonella dispar*, *Veillonella atypica*, *Veillonella rogosae*, *Veillonella denticariosi*, *Veillonella tobetsuensis*, and *Veillonella infantium*. These species vary in their distribution among different oral sites and among different patients (Luo et al., 2020).

### 5.2 *Veillonella* as a biomarker for lung cancer

In recent years, research has revealed that the *Veillonella* genus is associated not only with oral diseases but also with lung tumors. 16S rRNA amplicon sequencing has been utilized in numerous studies to investigate the oral microbiota, identifying early changes that may help identify lung cancer patients. Yan et al. conducted one of the earliest studies demonstrating the association between the saliva microbiome and lung cancer, observing high abundances of *Capnocytophaga* and *Veillonella* and lower abundances of *Neisseria* in the saliva of lung squamous cell carcinoma patients. These findings suggest that these bacteria could be potential biomarkers for disease detection (Yan et al., 2015). Hosgood et al. confirmed through a study involving 114 lung cancer patients and matched healthy controls that lower alpha diversity is linked to a higher risk of lung cancer (Hosgood et al., 2021). According to Yang et al., the saliva microbiomes of lung cancer patients are substantially less diverse and richer than those of healthy controls (Yang et al., 2018). Using droplet digital PCR technology, another study identified *Acidovorax* and *Veillonella* in Lung Squamous Cell Carcinoma patients, whereas Capophilic bacteria were related to lung adenocarcinoma, establishing *Acidovorax* as a biomarker that differentiates between the two primary forms of non-small cell lung cancer, SCC and lung adenocarcinoma (Leng et al., 2021). Additionally, in cohorts of 79 noncancer controls and 69 non-small cell lung cancer patients, the diagnostic value of *Acidovorax* and *Veillonella* as spit biomarkers for lung cancer was further validated (Leng et al., 2021). In addition, a mouse study revealed that *Veillonella parvula* is associated with various signaling pathways, including the IL-17, TNF-alpha, JAK-STAT, MAPK, and PI3K-AKT pathways (Tsay et al., 2021). A 2021 study revealed a correlation between the activation of the ERK and PI3K signaling pathways and the enrichment of *Streptococcus* and *Veillonella* in the lower airways of lung cancer patients (Tsay et al., 2018). Bacterial toxins such as LPSs and inflammatory cytokines released by immune cells play important roles in tumor development. However, the specific mechanisms of microbiome-mediated lung cancer progression remain unclear.

## 6 Anaerobic bacteria play a significant role in the occurrence and progression of tumors

### 6.1 Capnocytophaga

*Capnocytophaga* is a gram-negative anaerobic *bacillus* typically found in the oral cavity (Bilgrami et al., 1992). *Capnocytophaga* thrives in anaerobic environments containing carbon dioxide and exhibits gliding motility. Common species such as *Capnocytophaga ochracea*, *Capnocytophaga gingivalis*, and *Capnocytophaga sputigena* can cause infections in the oral cavity and other areas. Perera et al. reported that *Capnocytophaga* was overrepresented in OSCC patients compared with fibrous epithelial polyp controls (Perera et al., 2018; Stasiewicz and Karpiński, 2022). This result is in line with a study by Takahashi et al. that examined the oral microbiota of 60 patients with oral cancer and 80 controls and reported that the saliva of these patients contained more *Capnocytophaga* (Takahashi et al., 2019; Stasiewicz and Karpiński, 2022). Additionally, Q. Leng et al. reported high levels of *Capnocytophaga* and *Veillonella* in the saliva of lung squamous cell carcinoma patients, suggesting that *Capnocytophaga* could also serve as potential biomarkers for lung cancer (Leng et al., 2021).

### 6.2 Streptococcus anginosus

*Streptococcus anginosus* is a gram-positive, nonspore-forming, nonmotile bacterium that primarily resides in the oral cavity, nasopharynx, and gastrointestinal tract. It is known to cause invasive pyogenic infections. Research has revealed a strong correlation between *Streptococcus anginosus* infection and the emergence of gastric, esophageal, and oropharyngeal malignancies (Zhang W. L. et al., 2019). In a study of 42 cases of OSSC, *Streptococcus anginosus* DNA was detected in 19 cases (Sasaki et al., 2005). Morita et al. used real-time quantitative PCR to detect *S. anginosus* DNA in 8 cases of esophageal cancer and 38 cases of oral cancer, with 8 and 5 positive detections, respectively (Morita et al., 2003). *Streptococcus anginosus* may promote the development of oral cancer by damaging the oral mucosa, inducing chronic inflammation, and causing genetic alterations (Tateda et al., 2000). There is growing evidence that *Streptococcus anginosus* uses T-cell-mediated pathways to contribute to antitumor immunity. Early OSCC patients had a greater frequency of CD8^+^ T cells expressing granzyme B among all *Streptococcus* species detected, whereas late-stage OSCC patients presented an increased frequency of CD8^+^ T cells expressing granzyme B, particularly in response to *Streptococcus anginosus*. These findings indicate that Streptococcus-reactive CD8^+^ T-cell responses may contribute to antitumor immunity in OSCC patients (Wang et al., 2018). Additionally, increased Programmed Cell Death Protein 1 expression and Programmed death-1 expression are associated with lymph node metastasis and poor prognosis in oral cancer patients (Maruse et al., 2018). Further animal experiments are needed to elucidate the molecular mechanisms by which *Streptococcus anginosus* regulates OSCC development, antitumor immunity and prognosis. *Streptococcus anginosus* was recently identified by Jun Yu’s team as a “hidden culprit” in the promotion of gastric cancer.

Long-term infection in experimental mice rapidly induced acute gastritis, which then developed into chronic gastritis, mucosal metaplasia, and dysplasia, ultimately accelerating the development of gastric cancer. Researchers continue to explore related carcinogenesis mechanisms, revealing that *Streptococcus anginosus* can interact with the gastric epithelial cell surface proteins TMPC and Annexin A2, activating the MAPK signaling pathway. This interaction induces a precancerous process from superficial gastritis to atrophic gastritis and intestinal metaplasia, accelerating gastric cancer development, with an impact potentially comparable to *Helicobacter pylori* infection (Fu et al., 2024). Therefore, further research is urgently needed to determine the specific mechanisms by which *Streptococcus anginosus* infection leads to cancers beyond gastric and oropharyngeal cancers.

### 6.3 Other anaerobic bacteria

In addition to *Fusobacterium nucleatum* and *Porphyromonas gingivalis*, other anaerobic bacteria in the oral cavity are also considered potential carcinogens. These bacteria exhibit pathogenicity similar to that of *F. nucleatum* and *P. gingivalis*. Some anaerobic oral bacteria are extensively involved in tumorigenesis. Tannerella is a gram-negative anaerobic bacterium belonging to the Tannerella genus (Sharma, 2010). Tannerella is associated with other pathogens, such as *Porphyromonas gingivalis* and *Fusobacterium nucleatum*, and is a member of the red complex of periodontal pathogens. An elevated risk of esophageal cancer has been linked to T. forsythia. Through CD4^+^T helper cells and TNF-α, proinflammatory cytokines, such as IL-1β and IL-6, can be generated. glucose transporter 1 and glucose transporter 4 overexpression has been linked to enhanced tumor invasiveness in a variety of tumor types (Malinowski et al., 2019). In a study on the relationship between pancreatic cancer risk and oral microbiota, *Actinomyces* was found to be highly associated with pancreatic cancer, whereas *Leptotrichia* was associated with a lower risk (Fan et al., 2018). Additionally, genera such as *Dialister*, *Filifactor*, *Catonella*, and *Parvimonas* are highly involved in OSCC (Zhao et al., 2017). Recent research using 16S rRNA sequencing reported that *Aggregatibacter* is highly associated with laryngeal cancer, whereas *Bacteroides* and *Ruminiclostridium* are linked to a high risk of oropharyngeal cancer (Geng et al., 2019). Similarly, *Fusobacterium nucleatum* is also related to OSCC. Oribacterium levels are relatively high in the tongue coating of liver cancer patients, and Lautropia is considered a marker for ESCC (Sun et al., 2020). Currently, there are few studies on anaerobic bacteria causing related cancers, and more research and data support are needed to substantiate these findings. However, existing experimental evidence supports a close association between anaerobic bacteria and cancer development.

## 7 The anticancer potential of probiotics

Probiotics are a group of live microorganisms that can improve the host’s microbiome balance and provide definitive health benefits. They are widely colonized in the human gut, reproductive system, and oral cavity. The primary functions of probiotics include regulating the host’s microenvironment, promoting the production of beneficial metabolites, and inhibiting the colonization of harmful microorganisms. Common oral probiotics include *Bifidobacterium*, *Lactobacillus*, *Enterococcus*, *Saccharomyces*, *Bacillus*, and *Butyrivibrio*.

Studies have shown that many strains of *Lactobacillus* enhance intestinal barrier function, regulate immune tolerance, reduce microbial translocation in the gut mucosa, and alleviate gastrointestinal infections. Furthermore, probiotics play an important role in cancer prevention by inhibiting carcinogen effects, thus slowing the progression of cancer or tumor growth. The inhibitory metabolites produced by probiotics, such as organic acids (e.g., lactic acid, acetic acid), hydrogen peroxide, and bacteriocins (e.g., short peptides), are considered key factors in their anticancer activity.

*In vitro* studies have further revealed that butyrate and short-chain fatty acids produced by probiotics have significant anticancer effects. For example, *Lactobacillus paracasei* DG, *Lactobacillus johnsonii* CJ21, *Bacillus licheniformis* BL21 and *Bacillus subtilis* BS15 can exhibit antitumor and anti-proliferative effects by inhibiting histone deacetylase activity (Yu et al., 2024). Additionally, strains such as *Lactobacillus rhamnosus*, *Lactobacillus kefiri*, and *Lactobacillus acidophilus* induce apoptosis by regulating genes involved in cell homeostasis and proliferation pathways, further demonstrating their potential anticancer mechanisms (Dwivedi et al., 2021). Furthermore, some *Bifidobacterium* strains demonstrate significant anticancer properties through the production of enterolactones, while others inhibit the progression of leukemia in mouse models by fermenting linoleic acid to generate pectin oligosaccharides (Wei et al., 2018). It has also been demonstrated that adding Bifidobacterium longum subsp. infantis to soymilk inhibits the growth of the colorectal cancer cell lines HT-29 and Caco-2 (Wei et al., 2015). These results provide credence to the possible use of probiotics in the prevention and treatment of cancer.

## 8 Oral microbiota in clinical applications and progress

Recent research has demonstrated that the oral microbiota play a pivotal role in maintaining health and influencing disease pathogenesis, with their diversity often underestimated under normal physiological conditions. Antibiotics and cancer-related immunosuppression upset the delicate balance between commensal bacteria and the host in individuals with malignant tumors, leading to dysbiosis and worsening chemotherapy-induced oral mucositis (Dasari and Tchounwou, 2014; Wilson et al., 2014). Research has shown that chemotherapy patients have a significantly lower α-diversity of salivary microbiota, which is characterized by an enrichment of inflammation-associated Gram-negative taxa (like *Fusobacterium* and *Prevotella*) and a depletion of commensal taxa (like *Streptococcus* and *Actinomyces*) that are strongly correlated with the severity of mucositis (Ye et al., 2013; Hong et al., 2019).

Additionally, high doses of 5-fluorouracil and docetaxel have been shown to exacerbate dysbiosis in a dose-dependent manner (Hong et al., 2019). Modulating the microbiota, through approaches such as probiotics and microbiota-regulating agents, is considered an effective strategy for alleviating chemotherapy-associated mucositis, highlighting its potential in clinical diagnostics and therapeutics.

The study further clarified the distinctive changes in the oral and vaginal microbiota of patients with cervical cancer by using high-throughput 16S rRNA gene sequencing. These patients exhibited increased vaginal microbiota diversity with a significant reduction in *Lactobacillus* abundance, while oral microbiota diversity declined, accompanied by a marked enrichment of specific biomarkers such as *Fusobacterium* and *Prevotella* (Zhang et al., 2024). These microbiota may collectively contribute to the development and progression of cervical cancer through inflammatory and metabolic pathways. Diagnostic models based on these biomarkers have shown high sensitivity and specificity in cervical cancer screening. Furthermore, specific probiotics can effectively restore microbial balance and alleviate inflammation, laying the foundation for further clinical trials and the development of low-cost screening and personalized treatment strategies.

With the widespread adoption of 16S rRNA and metagenomic technologies, oral microbiota research has progressed from foundational microbial ecology to clinical applications, offering new perspectives for early disease diagnosis, precision therapy, and integrated prevention. Dynamic changes in oral microbiota have been identified as early diagnostic markers for conditions such as dental caries and periodontitis and are closely linked to systemic diseases (Lamont et al., 2018), including diabetes, cardiovascular diseases, and cancer (Matsha et al., 2020; Li et al., 2021). Interventions targeting oral microbiota, such as probiotic therapy and oral microbiota transplantation, are being explored to restore microbial balance and improve patient outcomes. Additionally, artificial microbiota models provide new avenues for investigating oral microbiota modulation.

In the future, the integration of artificial intelligence, big data analytics, and microbiome research is expected to enhance the role of oral microbiota studies in precision medicine. By utilizing cost-effective and efficient screening tools alongside personalized therapeutic strategies, oral microbiota modulation is poised to offer significant clinical value in disease management and health maintenance.

## 9 Discussions

One of the human body’s biggest sources of microbes is the oral cavity, and the association between the oral microbiome and cancer has become a highly focused topic. A growing body of research indicates a connection between cancer and the oral microbiome. Human health is greatly impacted by oral microorganisms, both within and outside of the oral cavity. These pathogens can directly or indirectly influence immune responses and affect normal cell signaling pathways before cancer onset. Well-known oral pathogens that can accelerate the progression of cancer include *Porphyromonas gingivalis* and *Fusobacterium nucleatum*. The mechanisms of bacteria-mediated carcinogenesis include the development of inflammatory conditions, immune suppression, and antiapoptotic activities. Through these mechanisms, oral bacteria can induce cancer. However, the development of cancer is a complex and prolonged process. Additional research is necessary to fully understand the causal link between oral bacteria and cancer. Research on the connection between other oral microbes and different types of cancer is still in its early stages; therefore, more investigations into the intricate processes by which bacteria affect the onset and spread of cancer are needed. However, current studies suggest that the abundance of cancer-specific oral bacteria could serve as a new clinical biomarker. Health assessments of deeper parts of the body through oral testing can lead to early predictions of certain types of cancer. Therefore, selecting accurate predictive indicators of cancer-related microbial variations and precise microbiological detection techniques is crucial. The use of oral microbiome detection for early cancer intervention and treatment could represent a significant breakthrough. Once the mechanisms of microbial carcinogenesis are fully elucidated, their application in treatment will be further explored and practiced. With the deepening integration of multidisciplinary approaches, oral microbiota research is expected to play a pivotal role in precision medicine, providing more efficient and cost-effective solutions for human health management. Moreover, microorganisms not only drive the development and progression of cancer but also mediate resistance to anticancer therapies. Whether antimicrobial treatments targeting cancer-associated microbiota can be clinically applied to significantly improve cancer patient survival and prognosis remains an area for further investigation.

## Glossary

GC: gastric cancer
CagA: Cytotoxin-associated gene A
ERK: extracellularsignal-regulated kinase
MAPK: mitogen-activated protein kinase
Bcl-2: B-cell lymphoma 2
CRC: Colorectal cancer
*F. Nucleatum*: *Fusobacterium nucleatum*
*P. Gingivalis*: *Porphyromonas gingivalis*
16S rRNA: 16S ribosomal RNA
FadA: *Fusobacterium* adhesin A
Fap2: Fibroblast Activation Protein-2
SH-2: Src homology 2
TCF: T-cell factors
LEF-1: Lymphoid enhancer-binding factor 1
TLR4: Toll-like receptor 4
PAK1: P21-activated kinases 1
NF-κB: Nuclear FactorKappa-B
IL-6: Interleukin 6
TNF-α: tumor necrosis factor α
TIGIT: T cell immune receptor with Ig and ITIM domains
OMV: outer membrane vesicles
TLRs: Toll-like receptors
TME: tumor microenvironment
MDSCs: Myeloid-derived suppressor cells
TANs: tumor-associated neutrophils
DCs: dendritic cells
TAMs: tumor-associated macrophages
CXCL: Chemokine (C-X-C motif) ligand
ESCC: esophageal squamous cell carcinoma
NLRP3: PYD domains-containing protein 3
BC: Breast Cancer
MMP-9: matrix metalloprotein 9
LPSs: lipopolysaccharides
*P.Gingivalis*: *Porphyromonas gingivalis*
OSCC: oral squamous cell carcinoma
PDCD4: programmed cell death 4
Fim: fimbriae
GECs: gingival epithelial cells
EMT: epithelial‒mesenchymal transition
IFN-γ: Interferon-γ
HSP27: Heat shock protein 27
MMPs: Matrix metalloproteinases
EMT: epithelial-mesenchymal transition
CDKs: cyclin-dependent kinases
SCC: squamous cell carcinoma
LSCC: laryngeal squamous cell carcinoma
